# The step‐and‐shoot IMRT overshooting phenomenon: a novel method to mitigate patient overdosage

**DOI:** 10.1120/jacmp.v17i4.6101

**Published:** 2016-07-08

**Authors:** Heming Zhen, Luo Ouyang, Qinan Bao, Nan Qin, Strahinja Stojadinovic, Arnold Pompos

**Affiliations:** ^1^ Department of Radiation Oncology Rush University Medical Center Chicago IL; ^2^ Department of Radiation Oncology UT Southwestern Medical Center Dallas TX; ^3^ Department of Radiation Oncology Torrance Memorial Medical Center Torrance CA USA

**Keywords:** IMRT overshoot

## Abstract

The goal of this work is to evaluate the dosimetric impact of an overshooting phenomenon in step‐and‐shoot IMRT delivery, and to demonstrate a novel method to mitigate the issue. Five pelvis IMRT patients treated on Varian 2100C EX linacs with larger than +4.5% phantom ion chamber point‐dose difference relative to planned dose were investigated. For each patient plan, 5 fractions were delivered. DynaLog files were recorded and centi‐MU pulses from dose integrator board for every control point (CP) were counted using a commercial pulse counter. The counter recorded CP MU agrees with DynaLog records, both showing an ~0.6 MU overshoot of the first segment of every beam. The 3D patient dose was recalculated from the counter records and compared to the planned dose, showing that the overshoot resulted in on average 2.05% of PTV $D_{95}$ error, and 2.49%, 2.61% and 2.45% of $D_{1\text{cc}}$ error for rectum, bladder, and bowel, respectively. The initial plans were then modified by inserting a specially designed MLC segment to the start of every beam. The modified plans were also delivered five times. The dose from the modified delivery was calculated using counter recorded CP MU. The corresponding $D_{x}$ parameters were all within 0.31% from the original plan. IMRT QA results also show a 2.2% improvement in ion chamber point‐dose agreement. The results demonstrate that the proposed plan modification method effectively eliminates the overdosage from the overshooting phenomenon.

PACS number(s): 87.55.Qr, 87.55.km

## I. INTRODUCTION

Intensity‐modulated radiation therapy (IMRT) has become widely used for a variety of clinical indications during the past decade. The inverse planning algorithm and the intensity modulation using multileaf collimator (MLC) have facilitated high‐quality radiotherapy planning and delivery. A key factor for dosimetric accuracy of delivered IMRT treatment fields is the coordination between leaf positions and monitor unit (MU) output. In step‐and‐shoot IMRT delivery, for example, the accuracy of the delivered MU of each individual MLC segment is limited by the frequency that the MLC controller checks the cumulated MU and the system lag for actually halting the radiation beam, causing a so‐called “overshoot” phenomenon.

This phenomenon was first described by Ezzell et al.^(1)^ for the 2100C series linac (Varian Medical Systems, Inc., Palo Alto, CA). Ezzell and colleagues reported that the control loop of Varian DMLC system (V4.8) requires 65 ms to monitor and halt the radiation of an MLC segment. As a result, the first segment will always deliver more MU than planned. The middle segments were assumed to deliver the correct MU if the overshoot is constant for each segment. The last segment of each field always undershoots because it is stopped by the MU1 signal which accurately controls the total MU. This phenomenon was also demonstrated by Xia et al.(2) with artificially created simple MLC geometry. Both studies suggested that the absolute amount of overshooting increases as dose rate increases, and that the relative error decreases with increasing the prescribed MU. At dose rate of 600 MU/min about 0.6 MU overshoot was observed for the MLC controller with 50 ms (20 Hz) cumulated MU checkpoint frequency. However, patient dose error was not quantified in these initial studies and both groups suggested that the clinical dose errors may be insignificant, especially in high‐dose regions.

Grigorov et al.^(3)^ and Kuperman et al.^(4)^ studied the impact of the overshoot phenomenon for site specific clinical cases. Grigorov and colleagues manually subtracted 0.65 MU, while Kuperman et al. subtracted 0.5 MU from the first segment then added the same MU number to the last segment, and recalculated the patient dose utilizing treatment planning system (TPS). For head and neck (HN) treatments, the Kuperman study found a 1%–2% overshoot in terms of minimum and maximum dose in both target and normal tissue. For prostate treatments, Grigorov et al.^(3)^ found 0.5 Gy increase in rectum mean dose due to overshoot phenomenon, which corresponded to 2%–3% increase in NTCP. Both authors proposed their remedy for this issue. Grigorov et al. suggest manually subtracting/adding a fixed amount of MU to the first/last segment in the treatment plan. Kuperman et al. proposed alternating the order of the segments in each beam through the treatment fractions.

However, the exact amount of overshoot/undershoot of the first/last segment is not a fixed number. The effect is a function of the prescribed segment MU and the actual delivery dose rate. Additionally, the middle segments MUs are not always delivered correctly, as assumed by Ezzell et al.,^(1)^ but rather have a random error. The net effect of this phenomenon depends on the exact ΔMU of the overshoot/undershoot of each segment, as well as the shape of these segments and patient geometry. The exact delivered segment MUs are needed to accurately quantify the effect of this phenomenon on patient dose. Simply subtracting/adding a fixed amount of MU may not accurately compensate the delivery error.

When analyzing the IMRT QA data from our institution, we have identified a pattern of unusually high discrepancies in a subset of patients due to the overshoot phenomenon. This was particularly notable for pelvis step‐and‐shoot treatments utilizing relatively large IMRT fields where ion chamber dose measurements were more than 4.5% higher than those predicted by TPS. We proved that this was happening due to the step‐and‐shoot overshoot phenomena. We thoroughly investigated the impact of this phenomenon on patient dose/DVH using MLC DynaLog files (Varian Medical Systems) and a pulse counter. As a result, we established a novel method to mitigate this problem, and experimentally validated the proposed solution.

## II. MATERIALS AND METHODS

### A. Treatment plan selection

Five clinically approved and irradiated pelvic step‐and‐shoot IMRT patients were selected for this study. All five clinical treatment plans were created in Pinnacle v.9.2 (Philips Radiation Oncology Systems, Fitchburg, WI) TPS, and delivered using Varian 2100C‐EX linacs (Varian Medical Systems, Palo Alto, CA) equipped with 120‐leaf Millennium MLC. The plans were calculated using the collapsed cone convolution dose algorithm in heterogeneous mode. The optimization was performed utilizing the direct machine parameter optimization (DMPO) algorithm using 15 beams. The IMRT parameters were set to maximum 50 segments per beam, $4{\text{cm}}^{2}$ minimum segment area, minimum 3 MUs per segment, and a $4\times 4\times 4\;{\text{mm}}^{3}$ calculation grid. For all five patients, the initial IMRT QA using ion chamber and Solid Water phantom (Gammex RMI, Middleton, WI) setup revealed point dose error higher than 4.5%. A closer investigation of these plans showed that for each individual beam, the first segment aperture always covered nearly the entire desired irradiated area (usually the largest segment), while the last segment mostly used very small aperture. The majority of the segments' dose contribution was small with only 3–5 MUs per aperture.

### B. The magnitude of the overshoot phenomenon

#### B.1 Quantification of segment output error

DynaLog files contain recorded MLC positions and fractional MUs per segment for every beam delivery. The fractional MU information is given relative to the meterset of 25,000 for the whole beam, regardless of number of segments used in the beam. Independent verification of the recorded MUs in DynaLog files requires the ability to physically count pulses independently from linac electronic circuitry. Such a device is indeed called a pulse counter. We used a BK1856D (B&K Precision Corp. Yorba Linda, CA) pulse counter which was connected to the linac dose integrating board to precisely record the number of centi‐MU pulses for every control point. This procedure serves to cross‐validate the recorded DynaLog files' MUs and the functionality of the pulse counter.

Each selected patient plan was repeatedly delivered five times, at a dose rate of 600MU/ min. For each delivery, the DynaLog files were saved and analyzed. Next, the delivered segment MU was compared to the planned segment MU to determine ΔMU for each segment and each delivery. The data were used for QA analysis and patient dose reconstruction.(^5–8^) Lastly, statistical analysis was performed to quantify the magnitude of overshooting net effect.

#### B.2 Quantification of clinical dose/DVH error

In order to find the clinically relevant patient dose error caused by the overshoot phenomenon, we recalculated the patient dose based on the centi‐MU counter records. For each patient plan, the TPS plan file which contains the detailed MLC positions and segment MUs was extracted from Pinnacle (Philips Healthcare, Andover, MA). An in‐house developed MATLAB code (MathWorks, Natick, MA) was used to overwrite the planned segment MUs in this file with the pulse counter‐recorded delivered segment MUs, creating a “delivered” plan file. The delivered plan file was then reimported to Pinnacle and patient dose was recalculated within TPS. The recalculated “delivered” patient dose was compared to the original TPS plan by evaluating the percent dose difference of every voxel. Furthermore, clinical DVH criteria were also evaluated, including the dose PTV $D_{95}$, rectum $D_{1\text{cc}}$ and $V_{50}$, bladder $D_{1\text{cc}}$ and $D_{\text{mean}}$, and bowel $D_{1\text{cc}}$ and $V_{15}$.

### C. The effect of adding MLC closed segments

As a general rule, there are two signature features that are intrinsically linked with the overshoot phenomenon. Feature one is that the first segment of every beam will undergo systematic overshoot, the last segment will undergo systematic undershoot, and the middle segments will have random variations that will be averaged out through the treatment fractions. Signature feature number two is that the first segment always has the largest aperture while the last segment is mostly very small, as the step‐and‐shoot optimization algorithm is designed to first fill the majority of the field with a uniform low intensity, and then paint small areas with higher intensity. As a consequence, the net dosimetric effect is predominantly determined by the overshoot of the largest first segment because it cannot be compensated by the undershoot of the last small segment.

In order to mitigate the MU overshooting of the first segment, a “closed segment” was added before the first clinical segment of each beam. The “closed segment” consisted of a small MLC aperture of $0.5\times 1\;{\text{cm}}^{2}$ hidden under the jaws and it was assigned with 1 MU. The “closed segment” cannot be literally completely closed because such a segment would not be recognized as a valid MLC segment. Moreover, the last segment of each clinical beam was examined for its aperture size. If the last segment had a large aperture (e.g., more than 80% of the entire irradiated area), an additional “closed segment” was added after it to mitigate the undershooting of the last clinical segment. The principal idea is to “transfer” the overshoot/undershoot effect from the first/last clinical segments to these “closed segments” that deliver virtually zero dose to a patient and, therefore, greatly reduce the net dosimetric impact of such an effect. An in‐house MATLAB code was used to insert these “closed segments” into the clinical plan file from Pinnacle TPS, creating a modified plan file for each patient. These modified plan files were then imported back to Pinnacle TPS, and the patient dose was recalculated. Obviously recalculating the dose introduced negligible changes, since only MLC transmission and leakage for 2 MUs were added. The modified plans were exported to record and verify system (MOSAIQ, Elekta, Sunnyvale CA) for delivery.

For each patient, the modified plan was delivered five times. The centi‐MU signals of the modified delivery were recorded using the pulse counter, as mentioned in section B above. The delivered MUs for each segment were extracted from the pulse counter records and compared to the clinically planned segment MUs. The original plan file was edited using the actually delivered MUs to represent an exact delivery file. This file was reimported to Pinnacle, and the adjusted delivered patient dose was recalculated within TPS. The exact delivered voxel dose and patient DVHs were compared to the original clinical plan to evaluate the effectiveness of the solution.

**Figure 1:**
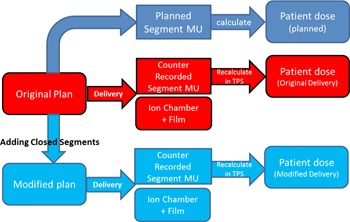
Flowchart of the study design.

In addition to recalculating using pulse counter record, IMRT QA measurements were performed for both original and modified delivery to verify the dosimetric improvement from the proposed plan modification. IMRT QA was delivered to solid water phantom with an ion chamber point to verify absolute point dose, and an EBT film to verify planar dose distribution. A flowchart that demonstrates the full experimental design is shown in Fig. 1.

## III. RESULTS

### A. Quantification of overshooting phenomenon

For all deliveries, the pulse counter recorded segment MUs agreed precisely with the DynaLog recorded MUs, except for the last segment of each beam. In fact, the DynaLog file for each segment records only the cumulative relative MUs, called the meterset value MS. For a particular segment i, the MU number associated with the ith segment ${\text{MU}}_{i}$ is given by

(1) $$MU_{i}=\frac{MS_{i}-MS_{i-1}}{25000}\cdot MU_{total}$$

where ${\text{MS}}_{i}$ is the cumulative meterset value after the ith segment, ${\text{MS}}_{i–1}$ is the meterset value for the (i‐1)th segment, ${\text{MU}}_{\text{total}}$ is total number of monitor units for a given beam, and 25,000 is the fixed total meterset value allocated for any beam. A detailed description of the DynaLog file can be found in the Varian reference guide.^(9)^

As it can be realized from Eq. (1), at the end of the last segment, the DynaLog file would report the value of 1 for every beam. The underlying implication is a flawless delivery (i.e., an error free scenario resulting in matched planned and delivered total number of MUs for each beam). However, the counter recorded last segment MUs for each beam was consistently higher than the one obtained from the DynaLog file by a few centi‐MUs. This is due to the time lag between the moment MU1 signal was generated and the instance when the radiation beam was actually terminated. The difference between counter recorded last segment MUs and the DynaLog record ranges from 0.03 MU to 0.09 MU, indicating a discrepancy of only of ~0.2% of the total MU number. This is also the difference between the delivered and planned MU of the beam. Based on this observation, we decided to perform the rest of the analysis using the counter recorded MUs to account for the small overshoot of the total MU.

**Figure 2:**
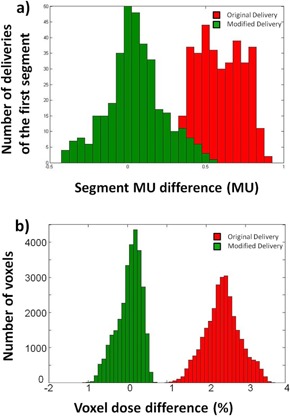
Histogram comparison of (a) first segment MU overshoot, and (b) voxel percentage dose difference, between the original delivery and the modified delivery.

Table 1 lists the ΔMU for the first segment, the middle segments, and the last segment for each patient plan, averaged over five repeated deliveries. It can be seen that the first segment has a constant overshoot of ~0.6 MU, the last segment has a constant undershoot of around ~0.6 MU, and the middle segment on average delivers the correct MU. Figure 2(a) shows the histogram (in red) of ΔMU for the first segment.

**Table 1:** The average value and range of ΔMU (difference between the delivered and planned segment MU) of the first, last, and a randomly selected middle segment, while delivering the original plan

	*First Segment*		*A Middle Segment*		*Last Segment*	
	*Average*	*Range*	*Average*	*Range*	*Average*	*Range*
Pt 01	0.60	(0.37, 0.87)	0.00	(‐0.51,0.46)	‐0.60	(‐0.84, ‐0.34)
Pt 02	0.59	(0.35, 0.87)	‐0.03	(‐0.43, 0.36)	‐0.56	(‐0.84, ‐0.36)
Pt 03	0.56	(0.33, 0.83)	‐0.04	(‐0.43, 0.42)	‐0.57	(‐0.86, 0.32)
Pt 04	0.62	(0.36, 0.89)	0.02	(‐0.45, 0.41)	‐0.61	(‐0.82, ‐0.34)
Pt 05	0.61	(0.37, 0.88)	‐0.02	(‐0.44, 0.49)	‐0.58	(‐0.92, ‐0.35)

**Figure 3:**
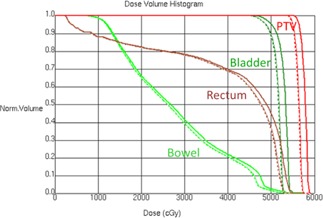
Comparison of the planned patient DVH and the delivered patient DVH for original delivery and modified delivery. Thin solid line; DVH from original plan; thick solid line: DVH from original delivery; dashed line: DVH from modified delivery.

Using the counter recorded segment MU information, we recalculated the patient dose and then compared it with the planned dose. A voxel‐by‐voxel local dose comparison was done for a high‐dose region that's encompassed by the 70% isodose surface. Figure 2(b) shows the histogram (in red) of the local percent difference of these voxels. In addition, the PTV $D_{95}$ and bladder $D_{1\text{cc}}$ parameters were also compared between the originally planned and the recalculated dose after the actual delivered MUs were taken into account. The percent differences between these dosimetric parameters are listed in Table 2. As indicated in Table 2, for the five tested patients, the overshoot phenomenon caused an overdose between 1.9% and 2.6% for all the $D_{x}$ parameters. Figure 3 shows the DVH comparison of the planned and delivered dose for a sample patient.

**Table 2:** The percent difference of selected dosimetric parameters between delivered and planned patient dose for both original delivery and modified delivery

	*PTV*				*Rectum*				*Bladder*				*Bowel*			
	$D_{95}$		$D_{50}$		$D_{1\text{cc}}$		$V_{50}$		$D_{1\text{cc}}$		$D_{\text{mean}}$		$D_{1\text{cc}}$		$V_{15}$	
	*Original*	*Modified*	*Original*	*Modified*	*Original*	*Modified*	*Original*	*Modified*	*Original*	*Modified*	*Original*	*Modified*	*Original*	*Modified*	*Original*	*Modified*
Pt 01	1.73%	0.31%	1.98%	0.40%	NA	NA	NA	NA	2.68%	0.52%	2.24%	0.52%	2.65%	0.54%	1.20%	0.13%
Pt 02	1.62%	−0.10%	1.88%	−0.04%	2.36%	−0.13%	NA	NA	2.52%	0.08%	2.38%	0.09%	1.64%	−0.24%	4.51%	0.10%
Pt 03	2.78%	−0.09%	2.65%	0.08%	2.77%	0.21%	21.44%	1.35%	2.90%	0.25%	2.80%	0.21%	2.62%	0.23%	1.50%	0.05%
Pt 04	2.23%	0.15%	2.50%	0.21%	2.71%	0.19%	NA	NA	3.00%	0.38%	3.32%	0.43%	2.31%	0.06%	1.10%	0.03%
Pt 05	1.91%	0.51%	2.74%	0.70%	2.12%	0.47%	15.16%	4.99%	1.97%	0.34%	1.74%	0.17%	3.04%	0.67%	1.33%	0.51%
Mean	2.05%	0.16%	2.35%	0.27%	2.49%	0.19%	9.15%	1.58%	2.61%	0.31%	2.49%	0.29%	2.45%	0.25%	1.93%	0.16%

### B. Evaluation of the modified delivery method

Figure 2(a) shows the histogram (in green) of ΔMU of the first clinical segment from the modified delivery after we inserted the closed segment. Note that the added closed segment significantly reduced the overshoot of the first clinical segment as the new segment displays a Gaussian‐like shape centered at around zero. In fact, the average ΔMU of the first clinical segment dropped to a negligible level of 0.02±0.18 MU.

The patient dose of the modified delivery after the closed segments were inserted was recalculated using the pulse counter recorded information and compared to the original clinical plan, the same way as described in the Materials & Methods section A. Figure 2(b) shows the histogram (in green) of the local percentage difference of the voxels in the high‐dose region, which is peaked at around zero, indicating the desired reduction of the overshooting phenomenon. The dosimetric parameters comparison showed that the modified delivery has reduced the dosimetric error to under 0.3% for the $D_{x}$ parameters. These numbers indicate the modified delivery is able to eliminate the systematic overshooting effect. Table 2 lists the percentage error for each patient. Figure 3 shows the DVH curve comparison of the modified delivery and the original clinical plan for a sample patient. Minimal difference was observed between the two set of DVH curves.

Table 3 lists the results from IMRT QA measurements for both original and modified delivery. It is shown that our plan modification improves the point dose difference from +5.2% to +3.0%. The magnitude of QA point dose improvement matches well with that of dosimetric indices ($D_{x}$) improvement of the PTV and OAR from pulse counter‐based calculation. The average gamma passing rate (3% global, 3 mm) for the original delivery was 80.1%, which was improved to 93.7% with the modified delivery.

**Table 3:** IMRT QA results for original and modified delivery

	*Point Dose Difference (%)*		*Gamma Passing Rate (%)*	
	*Original*	*Modified*	*Original*	*Modified*
Pt 01	4.7	3.4	72.8	91.6
Pt 02	6.0	4.2	87.9	92.3
Pt 03	5.9	3.2	81.0	94.2
Pt 04	3.9	1.5	88.3	96.0
Pt 05	5.4	2.7	70.5	94.6
Mean	5.2	3.0	80.1	93.7

## IV. DISCUSSION

In this work we reconstructed the patient dose using pulse counter recorded segment MU information from actual patient delivery. This procedure provides a more accurate assessment of the overshooting effect on patient dose compared to simply adding/subtracting a fixed amount of MU from the first/last segment, as it was performed in previous studies. Preceding published studies suggest that the overshooting should have an insignificant dose effect in the high‐dose region because the increased outputs do not tend to accumulate in the same voxels,^(1)^ and that the overdose to prostate PTV is less than 1%,^(3)^ while Kuperman et al.^(4)^ reported a 1%–2% overdose. Our cases show a higher dose impact: up to 2.8% in prostate PTV $D_{95}$ and 3.0% in bladder $D_{1\text{cc}}$. This is due to the special characteristics of these plans. In our cases, the first segment of each beam is always the largest and irradiates the whole target, while the rest of the segments are generally small and only irradiate a certain portion of the target. As a result, for a beam that delivers 60 MU, a 0.6 MU overshoot of the first segment may easily cause a more than 1% overdose. The 3.0% overdose in bladder $D_{1\text{cc}}$ that we observed is not insignificant, and could lead to increased bladder complication.

We found that, for any individual delivery, the ΔMU of the middle segments is not always insignificant, as indicated by the rather large range (‐0.51−0.49 MU) of the ΔMU of the middle segments in Table 1. The stochastic nature of these errors, however, causes random errors to average out, even with five deliveries. Our results strongly indicate that, for a customary fractionation scheme, excluding SRS or SBRT fractionations, the random errors on average have insignificant dosimetric impact.

There are several previously proposed methods to mitigate the overshooting effect. The simplest one is to reduce the dose rate. Based on our experience, dropping the dose rate from 600 MU/min to 300 MU/min will allow the point dose to pass our institutionally imposed IMRT QA criteria in most cases. In one extreme case, we had to change the dose rate to 100 MU/min to have the ion chamber measurement within tolerance. Although this may be the easiest solution, dropping the dose rate in theory would not eliminate the overshooting problem, yet it would significantly increases the treatment time. Grigorov et al.^(3)^ proposed a method to manually subtract/add a fixed number of MUs to the first/last segment. This method may not accurately correct for the overshooting unless the average amount of first/last segment overshooting/undershooting for each beam is known in advance. There will be a residual systematic error in segment MU if the added number differs from the true overshoot MU, which is not a constant if the segment has less than 5 MU. While our method shifts the unknown overshooting to an artificially added “closed segment” that essentially has no dosimetric impact, therefore completely removes the systematic error. Kuperman et al.^(4)^ proposed a different solution: shift the order of the segments for every fraction. This has the potential to completely remove the systematic error; however, it is relatively more complicated to implement because the number of fractions and the number of segment for each beam both varies, and the delivered plan needs to be changed for every fraction.

Other sources of error could also contribute to the rather large IMRT QA dose error for these large field IMRT deliveries. This is evidenced by the 3% ion chamber measurement results even after the plan modification. However, the goal of this study is to quantify and mitigate the contribution from the MU overshooting phenomenon. Recalculating patient dose using pulse counter record (cross‐validated with DynaLog files) allowed us to control other contributing factors. The magnitude of improvement from the plan modification was verified by IMRT QA measurement before and after modification.

The utilization of DynaLog files for patient‐specific treatment plan QA is becoming increasingly popular option amongst variety of clinical solutions. In our study, we cross‐compared the DynaLog record with the dose integrating board signal counted using a pulse counter. We found excellent agreement between the two methods in recording the delivered MU per segment. The only difference we saw was for the last segment because DynaLogs assume no error in the total MU for each beam. This result may serve as a validation of the MU output aspect of the DynaLog records for its use in treatment QA.

## V. CONCLUSIONS

The overshooting phenomenon caused by the system lag in Varian 2100C/EX machines could lead to 2.5%–3% overdose in PTV and normal tissues in selected pelvis step‐and‐shoot IMRT cases, as quantified through patient dose recalculation using counter recorded segment MU. We have proposed a novel method of adding a “closed” segment before the first clinical segment and possibly after the last clinical segment of each beam, and demonstrated through experiment that this method essentially removes the dosimetric effect of the overshooting phenomenon.

## COPYRIGHT

This work is licensed under a Creative Commons Attribution 3.0 Unported License.
